# Superior thermostability and divalent cation sensitivity of isoamylase CMI294C from *Cyanidioschyzon merolae*

**DOI:** 10.1007/s11103-025-01623-4

**Published:** 2025-07-31

**Authors:** Keisuke Okada, Taichi Someya, Takashi Osanai

**Affiliations:** School of Agriculture, Meiji University, 1-1-1 Higashimita, Tama-ku, Kawasaki, Kanagawa 214-8571 Japan

**Keywords:** Isoamylase, Semi-amylopectin, *Cyanidioschyzon merolae*, Thermostable diastatic enzyme

## Abstract

A storage polysaccharide in the red alga *Cyanidioschyzon merolae* is semi-amylopectin, a glucan with properties intermediate between noncrystalline glycogen and semicrystalline amylopectin. The debranching enzyme isoamylase plays a crucial role in determining the semicrystalline nature of glucans. In amylopectin-storing organisms, isoamylases consist of the isozymes ISA1, ISA2, and ISA3, with the former two primarily responsible for semicrystallinity. While the semicrystallinity of *C. merolae* semi-amylopectin is weaker than that of amylopectin, it retains a semicrystalline structure. Based on a previous analysis of isoamylase-deficient strains of *C. merolae*, the isoform CMI294C is the main contributor to glucan synthesis. Although the biochemical properties of isoamylases involved in amylopectin synthesis have been characterized, those of isoamylases involved in semi-amylopectin synthesis remain largely unknown. Here, we performed a detailed biochemical analysis of CMI294C to gain insights of isoamylases in semi-amylopectin synthesis. Similar to isoamylases in amylopectin-synthesizing organisms, CMI294C hydrolyzes amylopectin more efficiently than glycogen. However, unlike typical isoamylases, CMI294C is uniquely more active against pullulan than against glycogen; and it is strongly inhibited by Zn²⁺. Our results indicate that CMI294C can be potentially used for industrial maltose production due to its enzymatic properties. Overall, our findings provide molecular insights into the isoamylase in glucan structure modulation and enhance our understanding of glucan metabolism in *C. merolae*.

**Supplementary Information:**

The online version contains supplementary material available at 10.1007/s11103-025-01623-4.

## Key Message

CMI294C, the key isoamylase in *Cyanidioschyzon merolae*, exhibits unique properties, including strong Zn^²⁺^ inhibition and high pullulan activity, suggesting its role in glucan modulation and maltose production.

**Supplementary Information:**

The online version contains supplementary material available at 10.1007/s11103-025-01623-4.

## Introduction

Isoamylase (EC3.2.1.68; hereafter ISA) selectively hydrolyzes α-1,6-glycosidic linkages in glucans. It plays a critical role in the metabolism of branched glucans (e.g., glycogen and amylopectin) that are linked by α-1,4-glycosidic bonds in their linear regions and α-1,6-glycosidic bonds in their branched regions. Glycogen is characterized by short linear chains and lacks a crystalline region, whereas amylopectin has longer linear chains and a characteristic crystalline region. The crystalline region of amylopectin is formed through the alignment of linear chains with a degree of polymerization (hereafter DP) longer than DP10 and no intervening branch points, resulting in crystallization when two such linear chains form paired helices (Gidley and Bulpin 1987).

ISA has potential uses in the production of starch-derived sugars (e.g., glucose and maltose) in the saccharification industry. This process predominantly employs enzymes that hydrolyze α-1,4-glycosidic bonds, leaving α-1,6-glycosidic linkages in the branched regions intact. The consequent accumulation of limit dextrins reduce starch utilization efficiency (Li et al. 2023). Enzymes like ISAs that hydrolyze α-1,6-glycosidic bonds increase overall starch conversion efficiency (Li et al. 2023; Niu et al. 2022).

ISAs are expressed in all domains of life (bacteria, archaea, and eukaryotes). ISA is involved in amylopectin formation but not in glycogen formation. Three ISA isozymes, ISA1, ISA2, and ISA3, are involved in metabolizing amylopectin in photosynthetic organisms (Orzechowski 2008). Specifically, ISA1 and ISA2 are involved in amylopectin synthesis, while ISA3 is primarily involved in its degradation (Hussain et al. 2003). Functionally, ISA1 forms homo- or hetero-complexes with ISA2 to optimize the branching position of amylopectin and facilitate crystalline structure formation (Sim et al. 2014; Utsumi and Nakamura 2006). Disrupting this process produces phytoglycogen instead of amylopectin, leading to a glucan with low glucose density (Rahman et al. 1998). The red alga, *Cyanidioschyzon merolae*, accumulates a unique glucan called semi-amylopectin, which has intermediate properties between glycogen and amylopectin (Hirabaru et al. 2010). *C. merolae* has two ISAs, CMI294C and CMS197C, which are involved in the synthesis and degradation of semi-amylopectin, respectively (Matsuzaki et al. 2004; Maeno et al. 2022). Although it is known that CMI294C is essential for semi-amylopectin synthesis (Maeno et al. 2022), the extent to which the biochemical properties of ISAs involved in semi-amylopectin synthesis resemble those of ISAs involved in amylopectin synthesis remains unclear. This study aims to elucidate the molecular mechanisms underlying semi-amylopectin synthesis and glucan metabolism in *C. merolae* through a detailed analysis of CMI294C.

The saccharification industry requires enzymes that are both thermostable and acid-resistant. Proteins from *C. merolae* are known to be more thermally stable than those from other organisms (Ito et al. 2020; Rahman et al. 2017). A more detailed analysis of the thermostability and acid resistance of CMI294C is crucial for evaluating its potential value in industrial applications. Therefore, we characterized the properties of CMI294C in detail and explored its feasibility for use in the saccharification industry.

## Materials and methods

### CMI294C expression and purification

A vector containing both the gene encoding CMI294C and a GST-tagged fusion protein was introduced into *E. coli* BL21 competent cells (Takara Bio, Japan). Transformed cells were cultured in four Erlenmeyer flasks, each containing 400 mL LB medium at 37°C for approximately 21 h. One hour after the start of cultivation, 1.0 µL of 0.1 M IPTG (Wako Chemicals, Japan) was added to each flask to achieve a final concentration of 0.25 µM, inducing the expression of GST-tagged CMI294C. The cultured *E. coli* cells were collected by centrifugation (5,800×*g*, 2 min, 4℃), lysed by sonication (VC-750, EYELA, Japan), and centrifuged again (13,000×*g*, 2 min, 4℃) to separate the soluble and insoluble fractions. 1.0 mL of Glutathione Sepharose 4B resin (Cytiva, Japan) was centrifuged (5,800×*g*, 2 min, 4℃), and the supernatant was discarded. We then added enough PBS-T to the pellet to bring the volume to 1.5 mL, and the mixture was pipetted up and down. The mixture was then centrifuged (5,800×*g*, 2 min, 4℃), and the supernatant was discarded. This washing step with PBS-T was repeated three times. The washed resin was mixed with the supernatant of the bacterial lysate, and the GST-tagged protein was allowed to bind to the resin by gentle shaking on ice. After binding, the resin was collected by centrifugation (5,800×*g*, 2 min, 4℃), resuspended in PBS-T to 1.5 mL, and washed five times by repeating the centrifugation and supernatant removal. After the washes, we added 700 µL of GST elution buffer to the resin, mixed by pipetting, and centrifuged (5,800×*g*, 2 min, 4℃) the mixture. We collected 500 µL of the supernatant and added 500 µL of GST elution buffer to the resin, followed by pipetting and centrifugation (5,800×*g*, 2 min, 4℃). This elution step was repeated four times. The collected eluate containing GST-tagged CMI294C was concentrated using a Vivaspin 500 MWCO 50,000 spin column (Sartorius, Germany). We replaced the GST elution buffer by adding PBS-T to the column as the buffer level decreased during centrifugation (9,000×*g*, 4℃, various minutes). Finally, we collected GST-tagged CMI294C dissolved in 500 µL of PBS-T and confirmed protein purity by SDS-PAGE. The protein concentration was determined using the Pierce™ BCA Protein Assay Kit (Thermo Scientific™, USA).

### CMI294C activity assay

We used the dinitrosalicylic acid (hereafter DNS) method for quantifying reducing sugars to measure the activity of CMI294C (Bernfeld 1955). The reaction mixture (150 µL) consisted of 40 nM CMI294C, varying concentrations of soluble starch, and 100 mM MES-NaOH (pH 5.5−7.0). The mixture was incubated for 10 min at the specified temperature, then 750 µL DNS reagent was added. The mixture was incubated at 100°C for 5 min, cooled on ice for 5 min, and returned to room temperature. The reducing sugars were measured at 540 nm using a glucose standard curve to calculate the unknown concentrations.

We define one unit of enzymatic activity as the amount (µmol) of reducing sugar produced per minute. The reaction rate was calculated as units divided by the unit weight (mg) of CMI294C in the system.

### Assay of the effects of divalent cations

We assessed the effects of various additives on CMI294C activity by incorporating each one into the reaction mixture consisting of 40 nM CMI294C, 89 mg/mL soluble starch, and 100 mM MES-NaOH (pH 6.5). Divalent cations were selected based on the composition of the M-Allen medium (https://mcc.nies.go.jp/medium/ja/m_allen.pdf) used to culture *C. merolae*, and the corresponding metal salts were used.

### Assay of CMI294C thermostability

We analyzed the thermostability of CMI294C in a mixture consisting of a 250 µL of 133 nM CMI294C solution and 333 mM MES-NaOH (pH 6.5). The mixtures were heat-shocked at the indicated temperatures for 30 min followed by a cooling step on ice for 10 min. We then mixed 45 µL of the cooled samples with 105 µL of a 127 mg/mL soluble starch solution and incubated the enzymatic reaction at the same temperature as the heat shock for 10 min. Control samples consisted of enzymatic reactions that had not undergone heat shock treatment.

The half-life of CMI294C was determined using a 250-µL mixture of 133 nM CMI294C solution and 333 mM MES-NaOH (pH 6.5). The mixtures were heat-shocked at 60°C, 63°C, or 65 °C for 30, 60, 120, or 180 min followed by a cooling step on ice for 10 min. We then mixed 45 µL of the cooled samples with 105 µL of a 127 mg/mL soluble starch solution and incubated the enzymatic reaction at the same temperature as the heat shock for 10 min.

We evaluated the effect of Ca²⁺ on CMI294C thermostability using a mixture consisting of 80 nM CMI294C, 200 mM MES-NaOH (pH 6.5), and 2 mM CaCl_2_. The mixture was heat-shocked at 65℃ and 70℃ for 30 min followed by a cooling step on ice for 10 min. We mixed 75 µL of the cooled samples with 75 µL of a 178 mg/mL soluble starch solution and incubated each enzymatic reaction at the same temperature as the heat shock for 10 min.

### Assay of the CMI294C acid stability

We evaluated the acid stability of CMI294C using a mixture consisting of 80 nM CMI294C and sodium citrate buffer (200 mM citrate). The mixtures were incubated at various pH levels for 30 min at room temperature (22−26℃). Then 75 µL of the acid-treated samples was mixed with 75 µL of 178 mg/mL soluble starch solution, and the enzymatic reaction was incubated at 60℃ for 10 min. Control samples consisted of enzymatic reactions that had not undergone prior acid treatment.

### Examination of the substrate specificity CMI294C

We tested the activity of CMI294C against the following five substrates: soluble starch, starch (maize), amylopectin (maize), glycogen (oyster), and pullulan. Soluble starch was dissolved in heated ultrapure water, cooled to room temperature, and diluted with ultrapure water. Starch (maize) and amylopectin (maize) were suspended in hot water, treated with a sodium hydroxide solution (the final concentration at the substrate preparation is 500 mM), and heated with stirring for 20 min. Each mixture was cooled, neutralized with hydrochloric acid (adjusted to pH 6−7), and diluted with ultrapure water. Glycogen (oyster) and pullulan were dissolved in ultrapure water at room temperature. All enzymatic activity assays were performed using a mixture (150 µL) consisting of 15 µL of 400 nM CMI294C, 120 µL of varying concentrations of substrate, and 15 µL of 100 mM MES-NaOH (pH 6.5) at 55℃.

### Statistical analysis

We calculated all *P*-values from paired two-tailed Student’s *t*-tests using Microsoft Excel for Windows (Redmond, WA, USA). All experiments were conducted independently three times.

### CMI294C secondary structure prediction

We predicted the secondary structure of the amino acid sequence of CMI294C using NetSurfP 3.0 (https://services.healthtech.dtu.dk/services/NetSurfP-3.0/). We also compared the amino acid sequence of CMI294C to those of *Chlamydomonas reinhardtii* ISA1 (hereafter *Cr*ISA1) and *Pseudomonas amyloderamosa* ISA (hereafter *Ps*ISA), whose secondary structures were determined through crystallographic analysis, by ClustalW in MEGA11.

### Analysis of the three-dimensional structure

The amino acid sequence of CMI294C was submitted to AlphaFold (https://alphafoldserver.com) to predict its three-dimensional structure. To simulate interactions between CMI294C and various divalent cations (Mg²⁺, Ca^2+^, Mn²⁺, Zn²⁺, Co²⁺, Cu²⁺), simulations were performed using one molecule of CMI294C and one molecule of each cation under default settings. Three-dimensional structural data for the reference ISAs―*Cr*ISA1 and *Ps*ISA―were obtained from the Protein Data Bank (PDB; https://www.rcsb.org/), with PDB IDs 4OKD and 1BF2, respectively. For comparison with pullulanase, which exhibits high substrate specificity toward pullulan, we also used the structural data of *Hordeum vulgare* limit dextrinase (*Hv*LD; PDB ID: 4J3X) as a pullulanase. The coordinates of 6^3^-α-d-glucosyl-maltotriosyl-maltotriose (hereafter a branched maltoheptasaccharide) embedded in the substrate pocket of *Hv*LD were used for docking to each ISA. In this study, each glucose unit of the branched maltoheptasaccharide was labeled G1−G7 from the maltotriose end. Three-dimensional structural models were visualized and edited using CueMol2.

### Phylogenetic analysis of ISAs from red algae and amylopectin synthase-expressing organisms

We searched for sequences similar to CMI294C using BLAST to target ISAs from 11 organisms classified as red algae, green algae, and higher plants. Among the red algae, we selected all available sequences belonging to *C. merolae*, *Galdieria sulphuraria*, and *Chondrus crispus* that were registered in KEGG (https://www.genome.jp/kegg/). Among the green algae that synthesize amylopectin, we selected sequences from three—*Auxenochlorella protothecoides*, *Micromonas commoda*, and *Micromonas pusilla*—that are registered in KEGG and have multiple confirmed isozyme types. Among the higher plants that also synthesize amylopectin, we selected sequences from *Zea mays*, *Oryza sativa*, *Manihot esculenta*, *Solanum tuberosum*, and *Arabidopsis thaliana*, as they are model organisms in plant research with well-characterized ISA classifications in KEGG and biochemical analyses reported in previous studies. BLAST searches were performed within KEGG using the organism codes of the 11 selected organisms (in the order listed above: cme, gsl, ccp, apro, mis, mpp, zma, osa, mesc, sot, ath) against the amino acid sequence of CMI294C, using default settings. Among the identified ISAs, we selected all ISAs from red algae, whereas for green algae and higher plants, only ISAs with known isozyme types were chosen. All sequences were aligned using ClustalW in MEGA 11. The aligned sequences were analyzed by constructing a phylogenetic tree using the maximum likelihood method in MEGA 11, with 100 bootstrap replications.

## Results

### Purification and determination of the enzyme kinetics of GST-tagged CMI294C

**Fig. 1:**
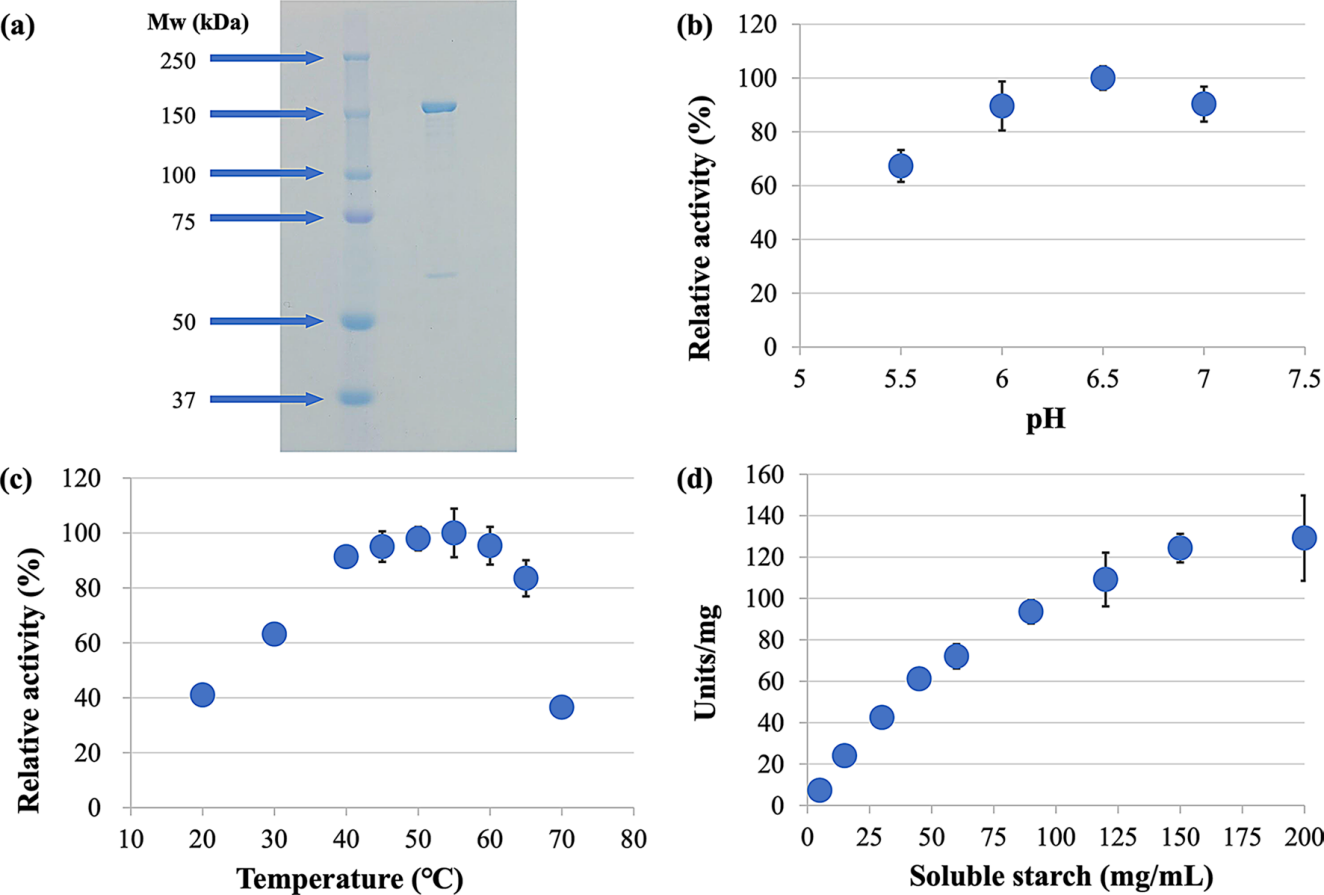
(**a**) SDS-PAGE analysis. The left lane contains the molecular weight marker, with the molecular weights of each band indicated by arrows. The molecular weight of the GST-tagged CMI294C is 136 kDa, comprising the GST tag (26 kDa) and CMI294C (110 kDa). The purified protein band is located adjacent to the 150 kDa band in the marker lane. (**b**) pH-dependent activity of CMI294C. Each point represents the mean, and error bars indicate the standard deviation (pH 5.5, *n* = 3; pH 6− 7, *n* = 6). The x-axis represents the buffer pH (MES-NaOH), and the y-axis shows the activity relative to the maximum activity of 100% at the optimal pH of 6.5. (**c**) Temperature-dependent activity of CMI294C. Each point represents the mean, and error bars indicate the standard deviation (20°C, 30°C, 70°C: *n* = 3; 40–65 °C: *n* = 6). The x-axis represents the reaction temperature, and the y-axis shows the activity relative to the maximum activity of 100% at the optimal temperature of 55 °C. (**d**) Substrate saturation curve of CMI294C. Each point represents the mean, and error bars indicate the standard deviation (*n* = 6).

Figure 1a shows the purified protein band adjacent to the 150-kDa marker band. We were able to recover 1640 µg of purified protein from 1.6 L of *E. coli* culture medium. CMI294C exhibited maximum activity at pH 6.5 and 55℃ (Fig. 1b c), and it retained over 90% of its relative activity across a wide temperature range (40−60℃). However, this activity sharply decreased to less than 40% at 70℃ (Fig. 1c).

We analyzed the reaction kinetics of CMI294C in response to various substrate concentrations under optimal conditions by measuring the activity of CMI294C against soluble starch at final concentrations ranging from 5 to 200 mg/mL (Fig. 1d). After calculating the reaction rates at each substrate concentration, we plotted a substrate saturation curve. The kinetic parameters were determined using both the Michaelis-Menten and Hill equations, the latter of which is particularly suitable for allosteric enzymes. The Michaelis-Menten model yielded the maximum reaction rate (*V*_*max*_) and substrate concentration at half-maximal velocity (*K*_*m*_) value of 209.9 ± 37.3 units/mg and 111.8 ± 23.5 mg/mL, respectively. The Hill equation yielded *V*_*max*_ and *K*_*m*_ values of 187.3 ± 39.1 units/mg and 89.0 ± 24.4 mg/mL, respectively. The *V*_*max*_ value derived from the Hill equation more closely matched the substrate saturation curve and was therefore adopted.

### Alteration of CMI294C activity by divalent cations

**Fig. 2:**
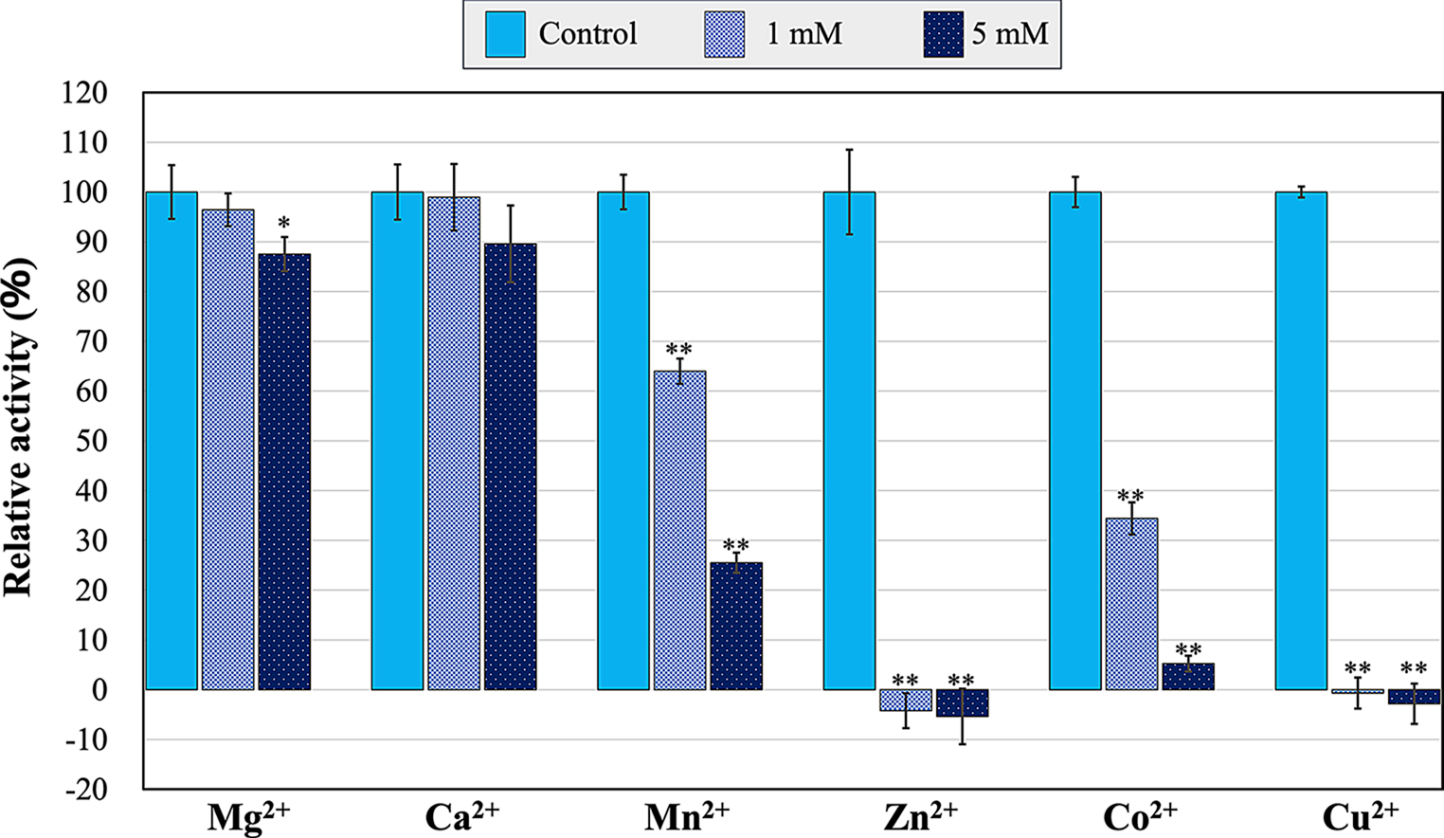
CMI294C activity in the presence of divalent cations. Each point represents the mean, and error bars indicate the standard deviation (*n* = 3). Activities are expressed relative to the control activity, which is set as 100%. *P < 0.05, **P < 0.005.

Because the activities of some ISAs are activated by calcium ions (Li et al. 2013; Lim et al. 2001; Panpetch et al. 2018; Duan et al. 2013), we investigated the effects of these and other divalent cations in the M-Allen medium on CMI294C activity. All the divalent cations tested reduced the activity of CMI294C (Fig. 2), although it retained more than 80% activity in response to 5 mM concentrations of the alkaline earth metal cations, Mg²⁺ and Ca²⁺ (Fig. 2). In contrast, the transition metal divalent cations (except for Mn²⁺ at 1 mM) reduced CMI294C activity to below 40% (Fig. 2). Notably, 1 mM concentrations of Zn²⁺ and Cu²⁺ caused complete inactivation of CMI294C (Fig. 2).

### Specificity of CMI294C for the hydrolysis of various substrates

We measured the reaction rates of CMI294C in response to various concentrations of five different substrates. At a substrate concentration of 45 mg/mL, the descending order of reaction rates were as follows: soluble starch > amylopectin (maize) > starch (maize) > pullulan > glycogen (oyster). Here, the relative activity against each glucan was calculated relative to the reaction rate against amylopectin, which was set as 100% (Table 1).

**Table 1:** Specificity of CMI294C for various substrates

Substrate	Relative activity (%)
Soluble starch	242.9 ± 17.3
Amylopectin (maize)	100.0 ± 12.6
Starch (maize)	56.2 ± 9.6
Pullulan	12.9 ± 1.3
Glycogen (oyster)	2.7 ± 0.3

The relative activity was calculated by setting the activity with maize amylopectin as 100%. Mean ± SD values for soluble starch, amylopectin (maize), and starch (maize) were calculated from six independent experiments. Mean ± SD values for pullulan and glycogen (oyster) were calculated from three independent experiments.

### High stability of CMI294C to high temperatures

**Fig. 3:**
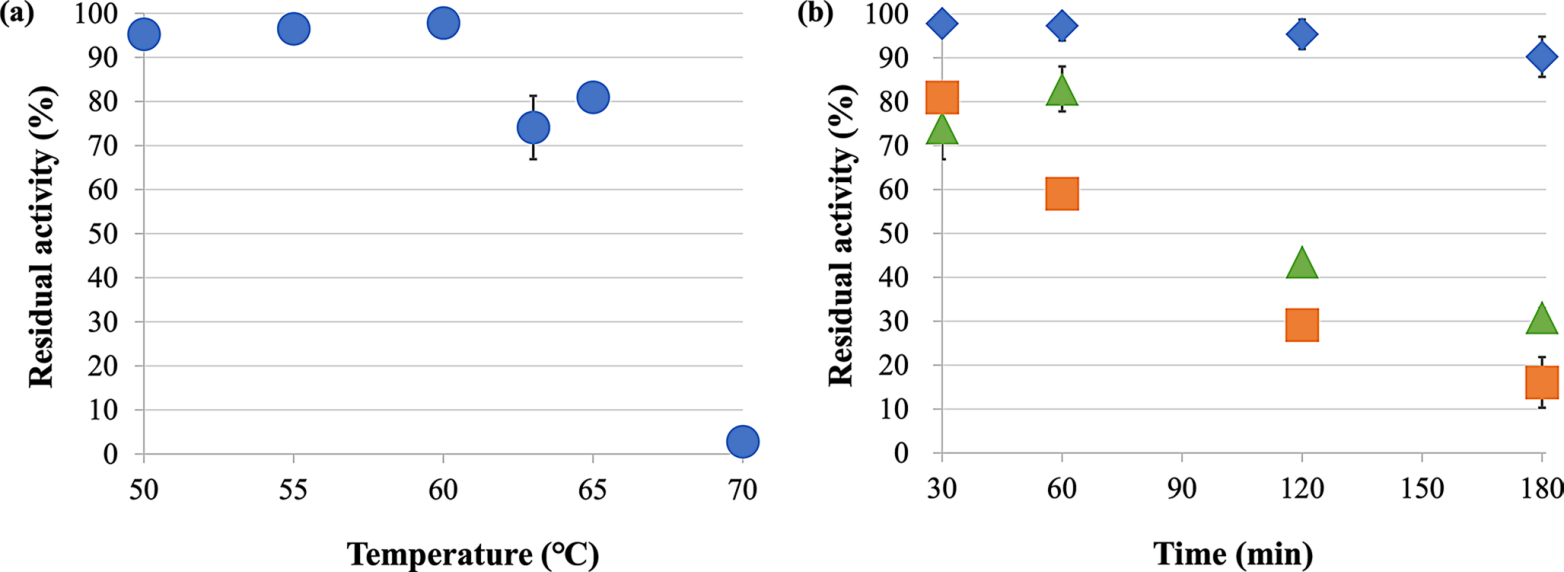
(**a**) Residual activity of CMI294C at various temperatures. Each point represents the mean, and error bars indicate the standard deviation (*n* = 3). The activity of the enzyme samples not subjected to heat shock treatment was set as 100%. (**b**) Time-dependent residual activity of CMI294C under heat shock treatment. Each point represents the mean, and error bars indicate the standard deviation (*n* = 3). The activity of the enzyme samples not subjected to heat shock treatment was set as 100%. Blue diamonds represent 60°C, green triangles 63°C, and orange squares 65 °C.

CMI294C heat-shocked (50–60°C) for 30 min retained more than 90% of its residual activity (Fig. 3a); however, a heat shock of 63°C or higher for 30 min resulted in the residual activity dropping to below 90%, and declining sharply to 2.8% after heat shock at 70°C (Fig. 3a). Prolonging the heat shock (60°C, 63°C, and 65°C) to 180 min resulted in residual activities of 90.2%, 30.9%, and 16.1%, respectively (Fig. 3b). We estimated the rate of activity loss by approximate linear regression to fit the residual activity data over time. Based on the regression equation, the half-life (*t*_1/2_) of the enzyme activity at each temperature are as follows: 1005, 120, and 89 min at 60°C, 63°C, and 65°C, respectively. The thermostability of CMI294C in the presence of Ca²⁺ was similar to that of the control at both 65°C and 70°C (Fig. S1).

**Fig. 4:**
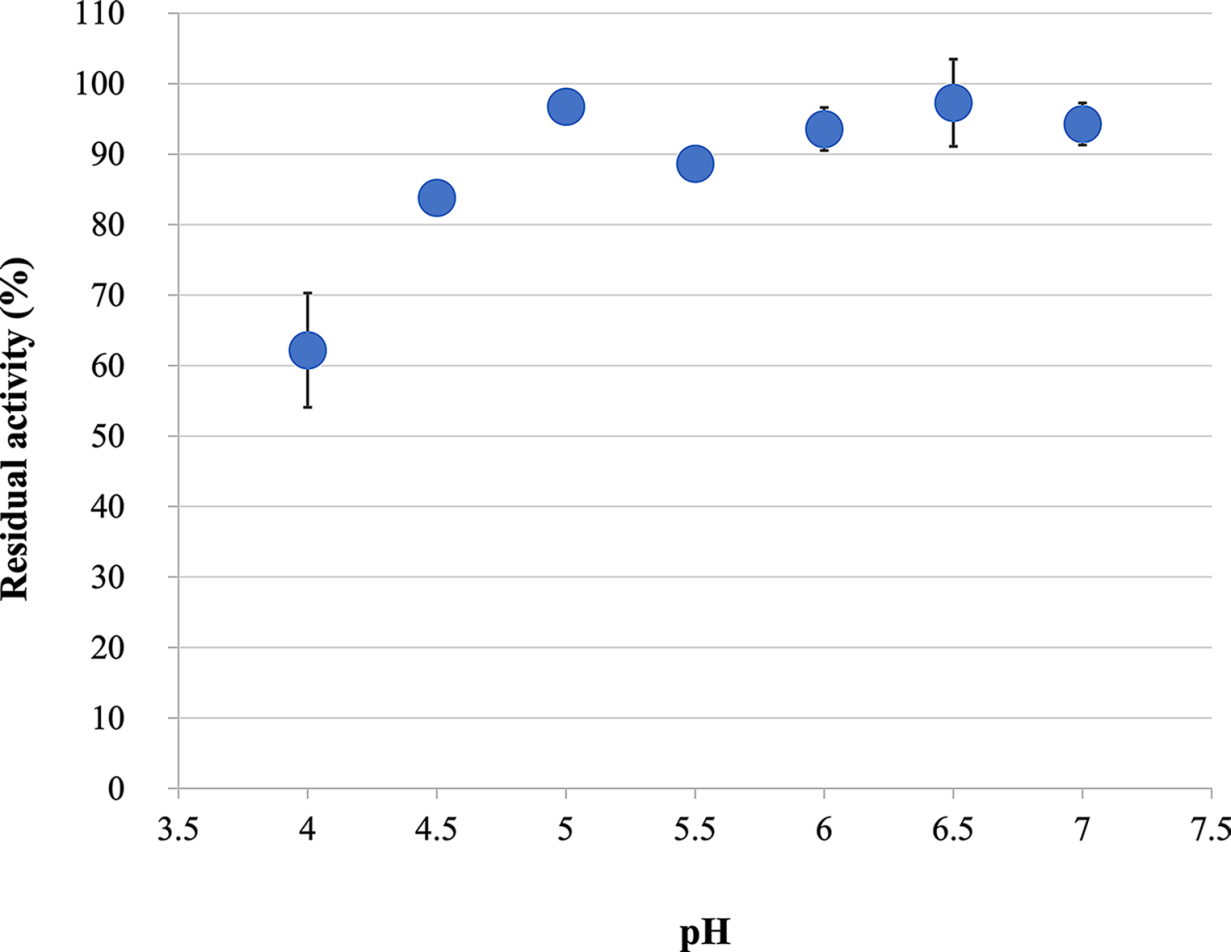
Residual activity of CMI294C across pH levels. Each point represents the mean, and error bars indicate the standard deviation (*n* = 3). The activity of the enzyme samples not subjected to acid treatment prior to the enzymatic reaction was set as 100%.

CMI294C retained was more than 80% of its activity between pH 4.5−7.0 (Fig. 4); however, below pH 5.0, its residual activity decreased as the pH decreased, and at pH 4.0, the residual activity was 62% (Fig. 4).

### Conserved structural features and unique sequence of CMI294C

We predicted the secondary structural features of CMI294C based on its sequence using NetSurfP 3.0. Fig. S2 shows the alignment results of the three sequences along with their respective secondary structures. The predicted structure of CMI294C resembled the secondary structure of *Cr*ISA1 and *Ps*ISA but differed in the presence of disordered residues in CMI294C. The following CMI294C residues were predicted disordered with greater than 90% probability: a.a.1−4 and a.a.836−1011. Comparison of the CMI294C sequence to that of *Ps*ISA indicates that the active site of CMI294C is conserved.

### Predicted structure and characteristics of CMI294C

Among the models predicted by AlphaFold, the local Distance Difference Test (hereafter plDDT) scores were below 50% for the N-terminal residues (a.a.1−38) and the C-terminal region (a.a.833−1011) (Fig. S3). In contrast, the central region (a.a.39−832), which had relatively high plDDT scores, exhibited a structure similar to *Cr*ISA1 and *Ps*ISA (Fig. S4). In divalent cation docking simulations, Mg²⁺, Ca^2+^, and Mn²⁺ were not predicted to bind any amino acid residues of CMI294C. Conversely, Zn²⁺, Co²⁺, and Cu²⁺ were predicted to bind to Cys393, with binding distances of 1.81 Å for Zn²⁺, 2.20 Å for Co²⁺, and 1.57 Å for Cu²⁺ (Fig. S5). Docking analysis of the branched maltoheptasaccharide into the substrate pockets revealed that six consecutive glucose units (G1−G6) were accommodated in CMI294C, three consecutive residues (G4−G6) in *Cr*ISA1, and three non-consecutive residues (G1, G2, and G6) in *Ps*ISA (Fig. S6). In all three ISAs, the catalytic residues were located near the α-1,6-glycosidic bond between G3 and G4, as indicated by dashed circles (Fig. S6).

### CMI294C grouped into a different cluster from higher plants and green algae

**Fig. 5:**
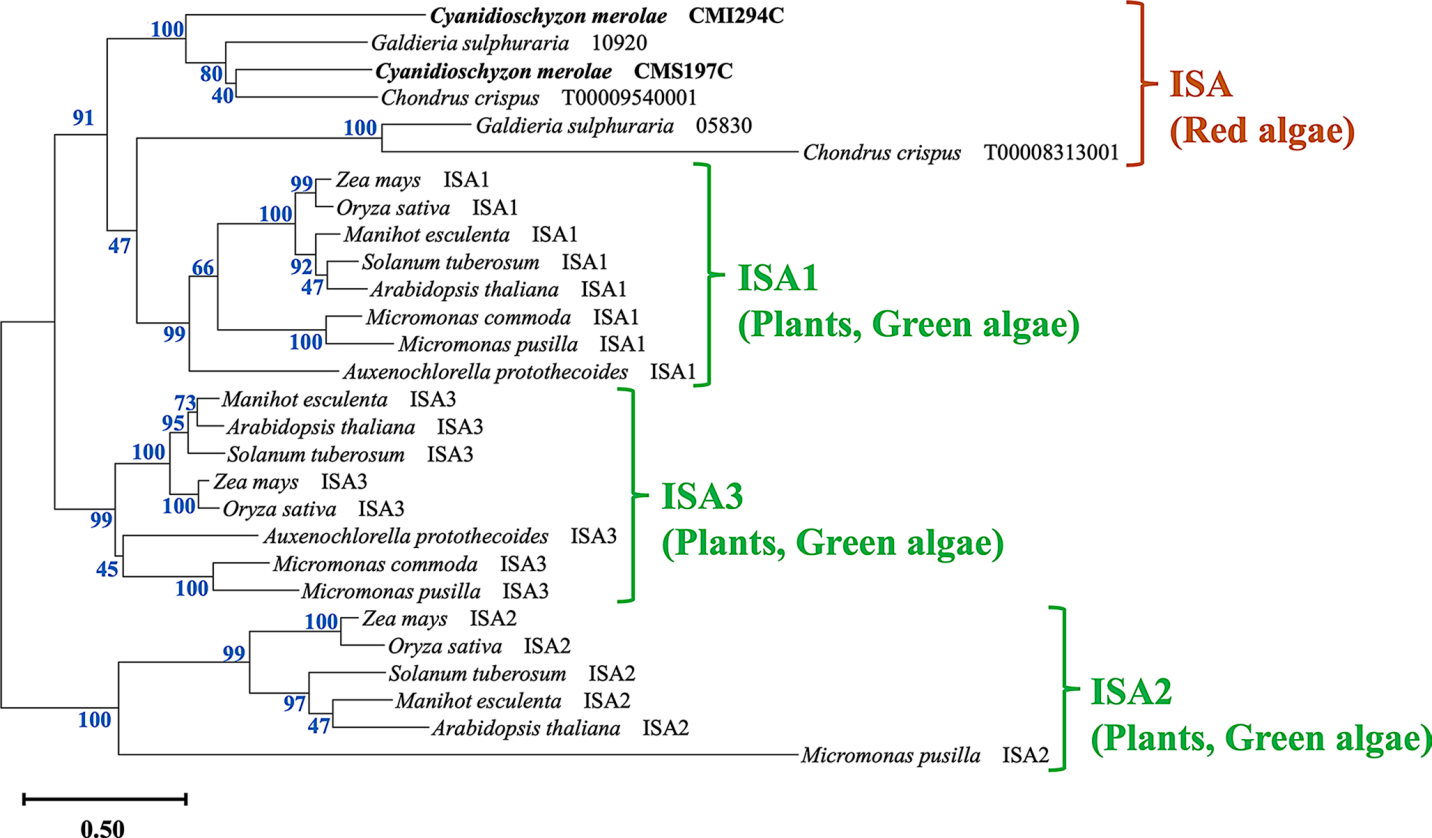
Phylogenetic tree of ISAs from red algae, higher plants, and green algae. The phylogenetic tree was constructed using the maximum likelihood method with 100 bootstrap replicates using MEGA 11. Blue numbers indicate bootstrap values that represent the probability(%) of reproducing each node in the constructed phylogenetic tree.

Phylogenetic analysis of CMI294C, with its distinctive sequence, and other eukaryotic photosynthetic organisms (Fig. 5) shows that ISA1, ISA2, and ISA3 comprise a phylogenetic group defined exclusively by their respective isozymes, regardless of higher plant or green alga origin. Meanwhile, CMI294C is grouped exclusively among ISAs from red algae (Fig. 5).

## Discussion

Our results indicate that the optimal activity of *C. merolae* ISA CMI294C occurs at pH 6.5, which falls within the intracellular pH range of *C. merolae* (pH 6.4−7.1) (Zenvirth et al. 1985). Similarly, CMI294C retained over 90% of its activity within the temperature range of 40–60°C, which includes 42°C—the optimal growth temperature of *C. merolae* (Kuroiwa et al. 2017). However, the optimal temperature for CMI294C activity is 55°C, which is the fifth highest among ISAs from 18 organisms; notably, it is the highest among the eight eukaryotic ISAs listed in Table S1.

The kinetic parameters of CMI294C and ISAs from other species (Table S2) show that the *V*_*max*_ of CMI294C for soluble starch exceeds that for any substrate tested for *O. sativa* ISA. In contrast, the *V*_*max*_ of *Bacillus* sp. KSM-3309 ISA is approximately 3.44 times higher than that of CMI294C for glycogen (oyster) and 3.14 times higher for amylopectin (potato). Meanwhile, *O. sativa* ISA cleaves extra branches during starch synthesis, while *Bacillus* sp. KSM-3309 ISA primarily degrades glucans. ISAs involved in starch synthesis likely have lower reaction rates to prevent excessive cleavage of the branching structures essential for starch formation. Therefore, we predict that CSM197C, which degrades semi-amylopectin, likely has a higher *V*_*max*_ than that of CMI294C (*V*_*max*_ = 187.3 ± 39.1 units/mg).

The substrate specificities of CMI294C and ISAs from various species (Table S3) show that the activity of CMI294C decreases in the following order: soluble starch > amylopectin (maize) > starch (maize) > pullulan > glycogen (oyster). Thus, CMI294C is least active against glycogen (oyster), even though this substrate has the second-highest α-1,6-glycosidic linkage density after pullulan. CMI294C selectively cleaves linear chains with a degree of polymerization (DP) of 12 or more among branched glucans (Maeno et al. 2022). Therefore, this may also explain the limited cleavage of glycogen (oyster) by CMI294C, because its average chain length is 11 (Matsui et al. 1996). This aligns with the glucan chain length analysis by Maeno et al. (2022). Although pullulan is generally resistant to ISA degradation (Kainuma et al. 1978), CMI294C was more active against pullulan than glycogen (oyster), suggesting a unique hydrolytic property. The substrate pocket of *Hv*LD, a pullulanase, can accommodate the entire pullulan fragment 6^3^-α-d-glucosyl-maltotriosyl-maltotriose (Møller et al. 2015). In contrast, the predicted structure of CMI294C and the known structures of *Cr*ISA1 and *Ps*ISA can only partially accommodate a branched maltoheptasaccharide, with CMI294C capable of accommodating the longest continuous stretch of six glucose units (Fig. S6). The broader and deeper substrate pocket of CMI294C likely contributes to its substrate specificity toward pullulan. Maize starch comprises approximately 73% amylopectin and amylose (Jane et al. 1992). Maize starch (60 mg/mL) contains approximately 44 mg/mL amylopectin. We compared the reaction rate of CMI294C with 60 mg/mL maize starch (19.1 units/mg; data not shown) to that with 45 mg/mL maize amylopectin (25.2 units/mg; data not shown). Therefore, the CMI294C activity on 60 mg/mL maize starch was 75.8% of the activity on 45 mg/mL maize amylopectin, suggesting that amylose inhibits CMI294C activity. Unlike ISAs from starch-synthesizing species such as *C. reinhardtii*, *Z. mays*, and *M. esculenta*, we confirmed no consistent preference for glycogen (oyster) or amylopectin (maize) among ISAs non-starch-synthesizing organisms.

The effects of cations on CMI294C and ISAs from various organisms are summarized in Table S4. Ca²⁺ increased the activities of ISAs from *M. esculenta*, *Pectobacterium chrysanthemi* PY35, *Thermobifida fusca*, and *Bacillus* sp. CICIM 304, whereas this cation did not enhance the activities of CMI294C and *O. sativa* ISA. CMI294C was strongly inhibited by Zn²⁺ and Cu²⁺, similar to ISAs from *P. chrysanthemi* PY35 and *Bacillus* sp. CICIM 304, which exhibited reduced activity to the former. In contrast, ISAs from *M. esculenta* and *T. fusca* were unaffected by Zn²⁺. Notably, CMI294C was inactivated at both 1 mM and 5 mM Zn²⁺, whereas other ISAs remained active. The inhibition of CMI294C by Cu²⁺ aligns with most ISAs, except that of *T. fusca*. Notably, *O. sativa* ISA was completely inactivated at 1 mM Cu²⁺, and *Bacillus* sp. CICIM 304 ISA lost activity at 0.5 mM Cu²⁺, indicating strong Cu²⁺ inhibition. We hypothesized that the inhibition of CMI294C by divalent cations occurs due to chelation by amino acid residues critical for activity. Three-dimensional simulations revealed that Mg²⁺, Ca^2+^, and Mn²⁺ did not interact with any residues, whereas Zn²⁺, Co²⁺, and Cu²⁺ were predicted to bind to the thiol group of Cys393, which is not a catalytic residue (Fig. S5). The predicted bond lengths between Cys393 and each cation followed the order: Co²⁺ >Zn²⁺ >Cu²⁺ (Fig. S5), which is consistent with the observed inhibitory effects, where Zn²⁺ and Cu²⁺ showed stronger inhibition than Co²⁺ (Fig. 2). Furthermore, when 5 mM EDTA was added to reaction mixtures containing 1 mM of each cation, the inhibition by Zn²⁺ and Co²⁺ was alleviated, whereas that by Cu²⁺ remained unchanged (Fig. S7). These results suggest that Zn²⁺ and Co²⁺ inhibit CMI294C via reversible chelation to amino acid residues critical for activity. Given that Cys393 is located close to the catalytic residues (Fig. S8), it may interact with other nearby residues important for function. By contrast, the irreversible inhibition by Cu²⁺ suggests a different mechanism, likely unrelated to chelation by protein residues.

The saccharification enzyme blend Extenda^®^ Standard (Novozymes, Denmark) for glucose production is recommended for use at 60–63 °C and pH 4.1−4.5, which are the optimal conditions for the main enzyme, glucoamylase (Niu et al. 2022). The half-life of CMI294C is 1005 min (16.8 h) under a heat shock at 60 °C, indicating sufficient thermostability at this temperature. However, its residual activity decreases at pH 4.0−5.0, indicating limited acid resistance in the optimal pH range for glucoamylase. Thus, despite its thermostability, CMI294C is unsuitable for glucose production due to its acid sensitivity. However, saccharification to maltose is typically conducted at 55−65 °C and pH 4.8−5.5 (Olsen 2002), which are favorable conditions for CMI294C, making it a promising enzyme for maltose production. The thermostability of CMI294C is unaffected by Ca²⁺. The α-amylases traditionally used in liquefaction processes require the addition of Ca²⁺ for optimal activity and stability (Machius et al. 1995), which often leads to calcium salt precipitation in pipes and tanks that necessitate frequent cleaning. In contrast, CMI294C does not require Ca²⁺ for either activity or stability, highlighting its potential usefulness in saccharification processes (Table S4; Fig. S1).

CMI294C shares structural features with *Cr*ISA1 and *Ps*ISA (Fig. S2). The active site of CMI294C comprises Asp427, Glu466, and Asp549. NetSurfP 3.0 predicts that a.a.836−1011 of CMI294C is more than 90% likely to consist of disordered residues, which typically lack a fixed conformation but can adopt a specific structure upon interaction with other molecules (Wright and Dyson 1999). However, the disordered status of the sequence a.a.836−1011 of CMI294C needs to be confirmed by structural analysis. If the disordered status is confirmed, then the structure of CMI294C a.a.836−1011 cannot be determined until further investigation to identify the molecules CMI294C interacts with.

Structural predictions indicated that the plDDT scores were below 50% for a.a.1−38 at the N-terminus and a.a.833−1011 at the C-terminus of CMI294C (Fig. S3). These findings are consistent with NetSurfP 3.0 predictions, which identified a.a.1−4 and a.a.836−1011 as disordered regions (Fig. S2). In contrast, the central region of CMI294C (a.a.39−832), which had higher plDDT scores, showed substantial structural similarity to *Cr*ISA1 and *Ps*ISA (Fig. S4). Moreover, the spatial arrangement of the catalytic residues was conserved among the three enzymes (Fig. S4). Taken together, these results suggest that the core region of CMI294C shares structural features with ISAs from other species, while the functional roles of the disordered N- and C-terminal regions remain to be elucidated.

CMI294C contributes to semi-amylopectin synthesis, while its isozyme CMS197C is involved in semi-amylopectin degradation (Maeno et al. 2022). Phylogenetic analysis clustered CMI294C with ISA1; however, a refined tree revealed that CMI294C and CMS197C form a distinct clade comprising only red algae-derived ISAs (Fig. 5). This suggests that their functional differentiation in *C. merolae* followed an evolutionary trajectory distinct from that of higher plants and green algae.

## Electronic supplementary material

Below is the link to the electronic supplementary material.

Supplementary Material 1

Supplementary Material 2

Supplementary Material 3

Supplementary Material 3

Supplementary Material 3

Supplementary Material 3

Supplementary Material 3

Supplementary Material 3

Supplementary Material 3

Supplementary Material 3

Supplementary Material 3

Supplementary Material 3

Supplementary Material 3

## Data Availability

Not applicable.
