# Supplementary figures and images for: Superior thermostability and divalent cation sensitivity of isoamylase CMI294C from Cyanidioschyzon merolae
Source: Plant Mol Biol. 2025 Jul 31;115(4):99. doi: 10.1007/s11103-025-01623-4 (PMC12313807; doi:10.1007/s11103-025-01623-4)

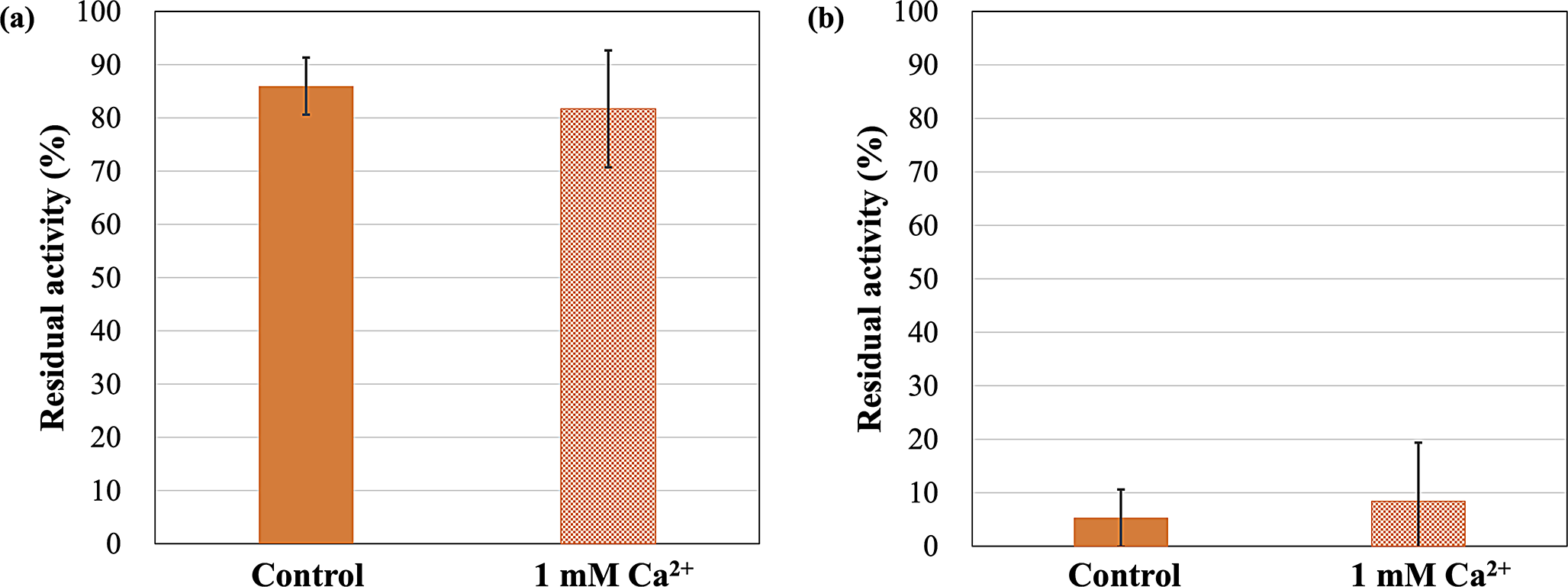

Supplement: Supplementary file 1 — Supplementary Material 1 [file 11103_2025_1623_MOESM1_ESM.tiff]

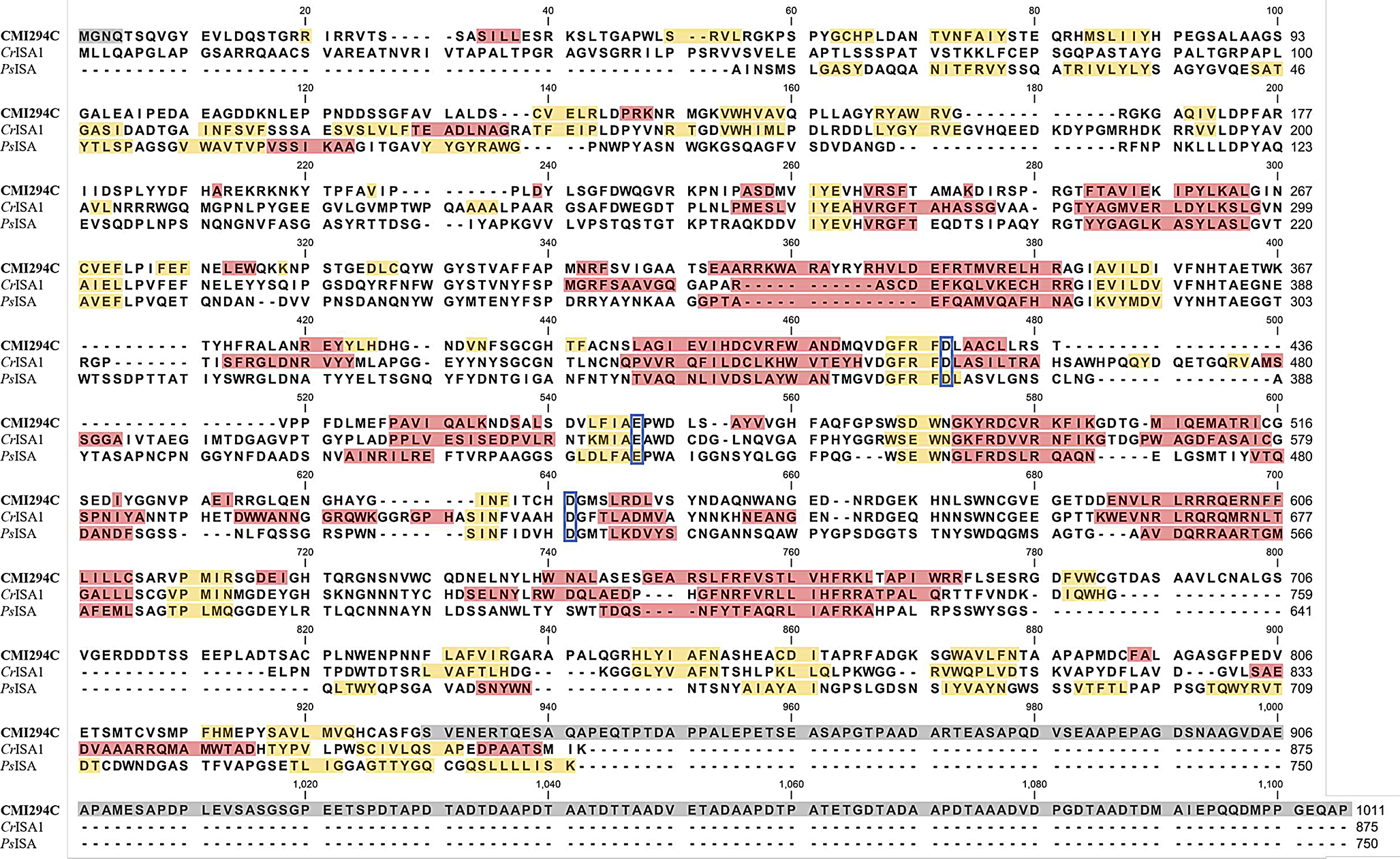

Supplement: Supplementary file 2 — Supplementary Material 2 [file 11103_2025_1623_MOESM2_ESM.tiff]

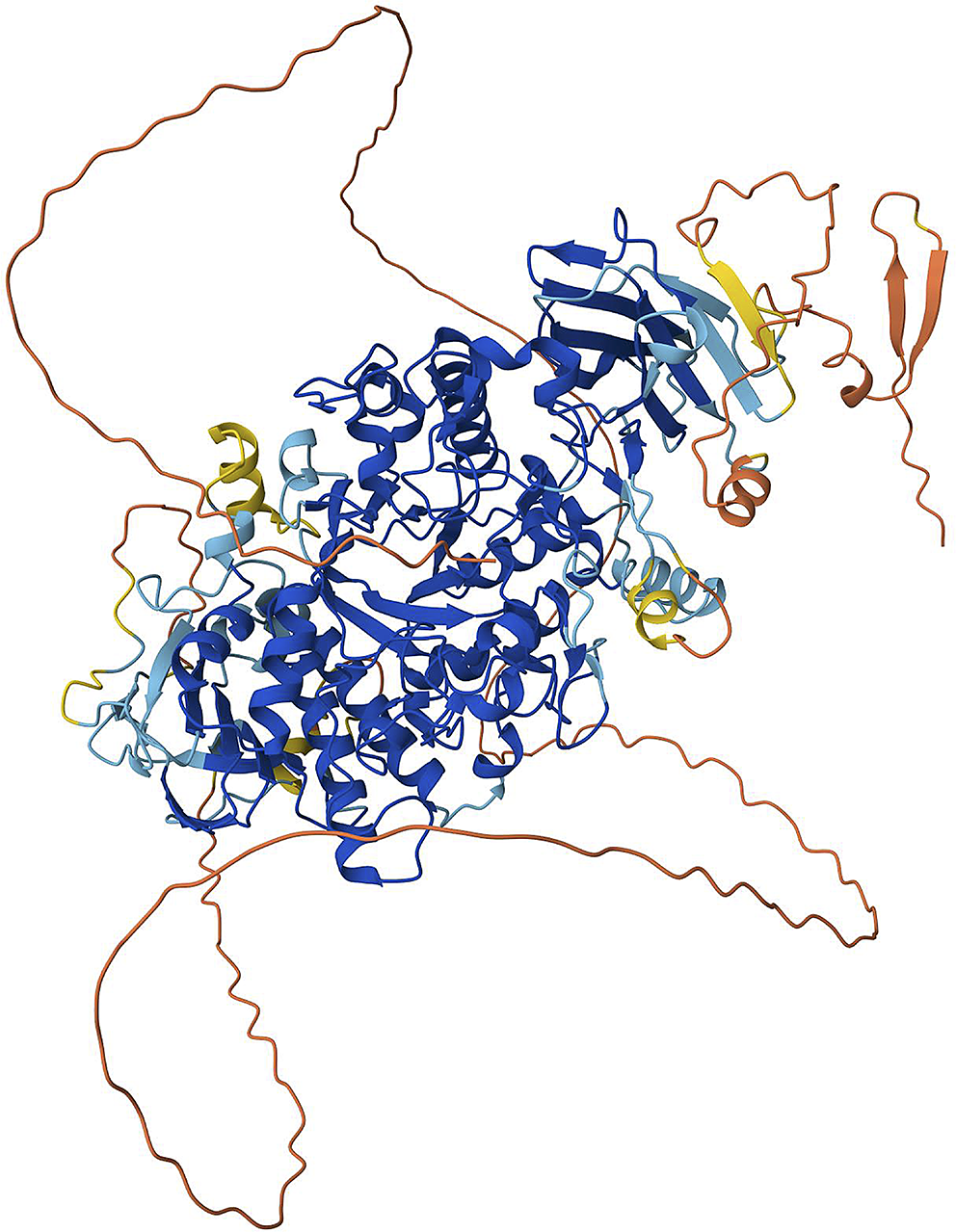

Supplement: Supplementary file 3 — Supplementary Material 3 [file 11103_2025_1623_MOESM3_ESM.tiff]

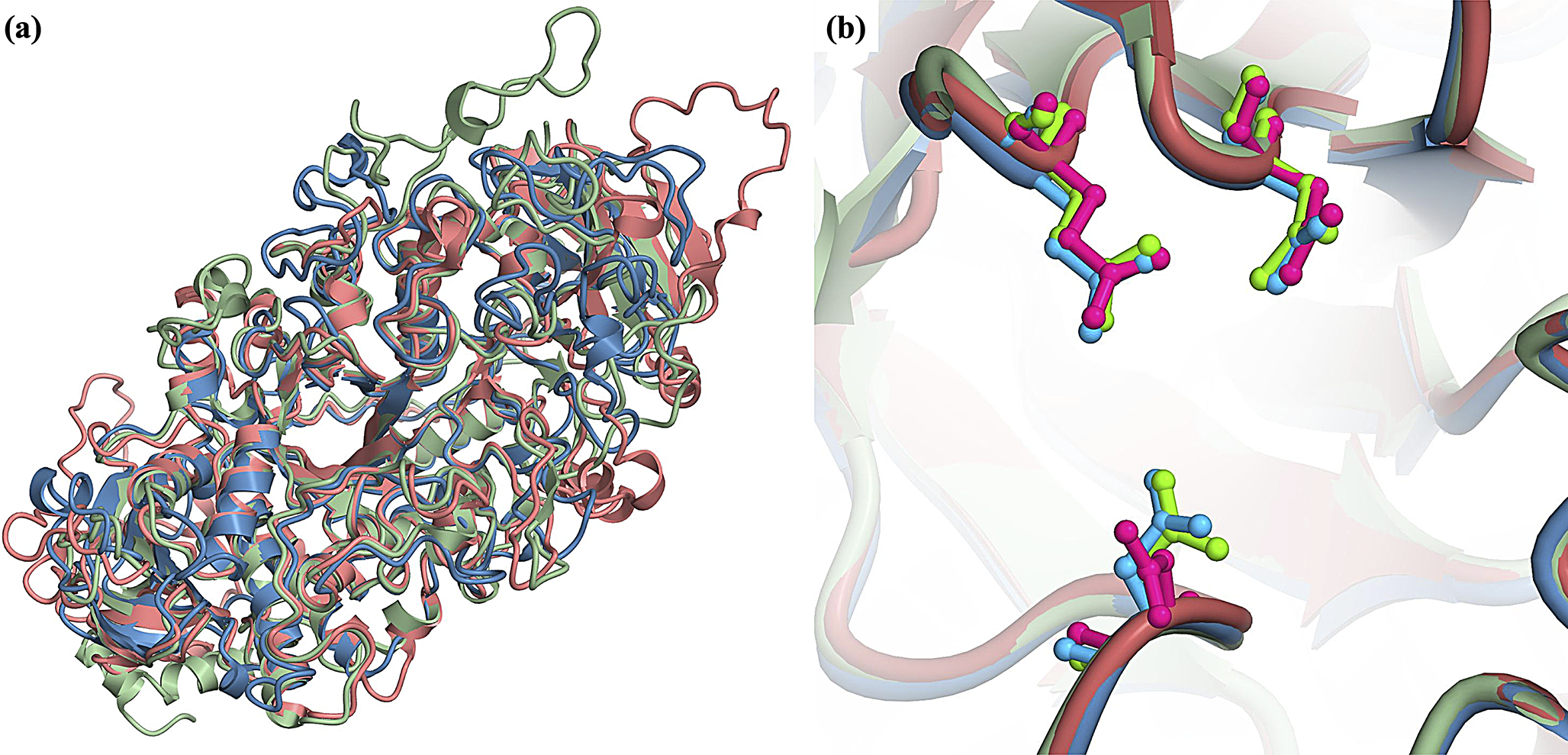

Supplement: Supplementary file 4 — Supplementary Material 3 [file 11103_2025_1623_MOESM4_ESM.tiff]

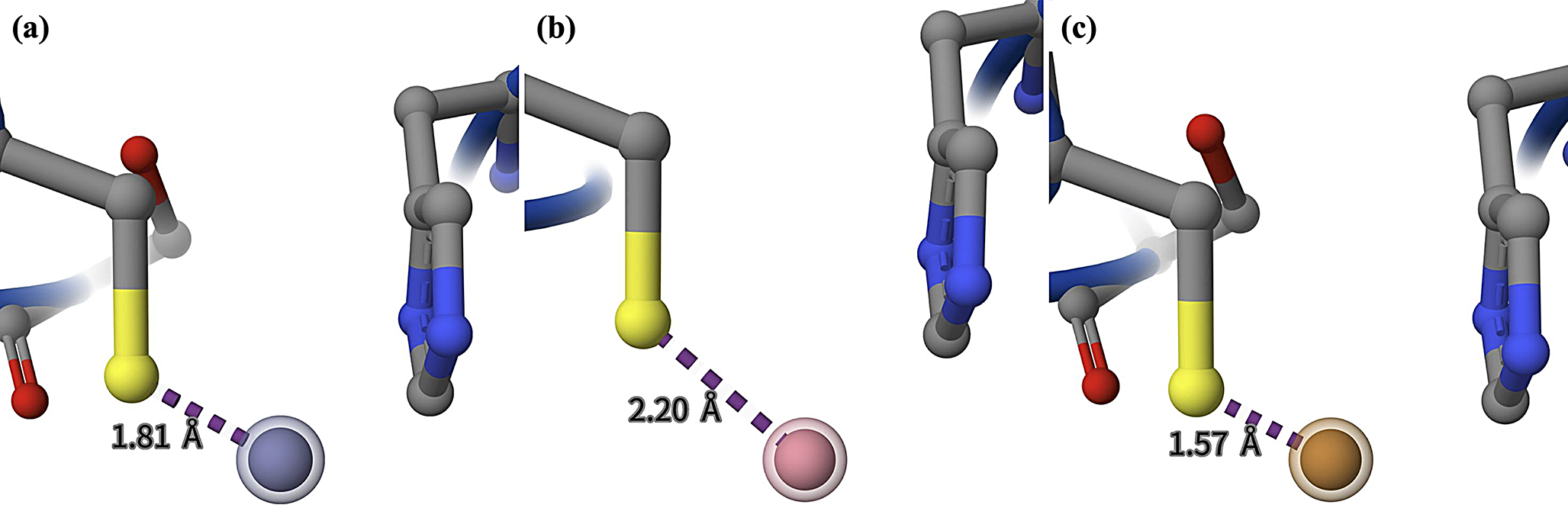

Supplement: Supplementary file 5 — Supplementary Material 3 [file 11103_2025_1623_MOESM5_ESM.tiff]

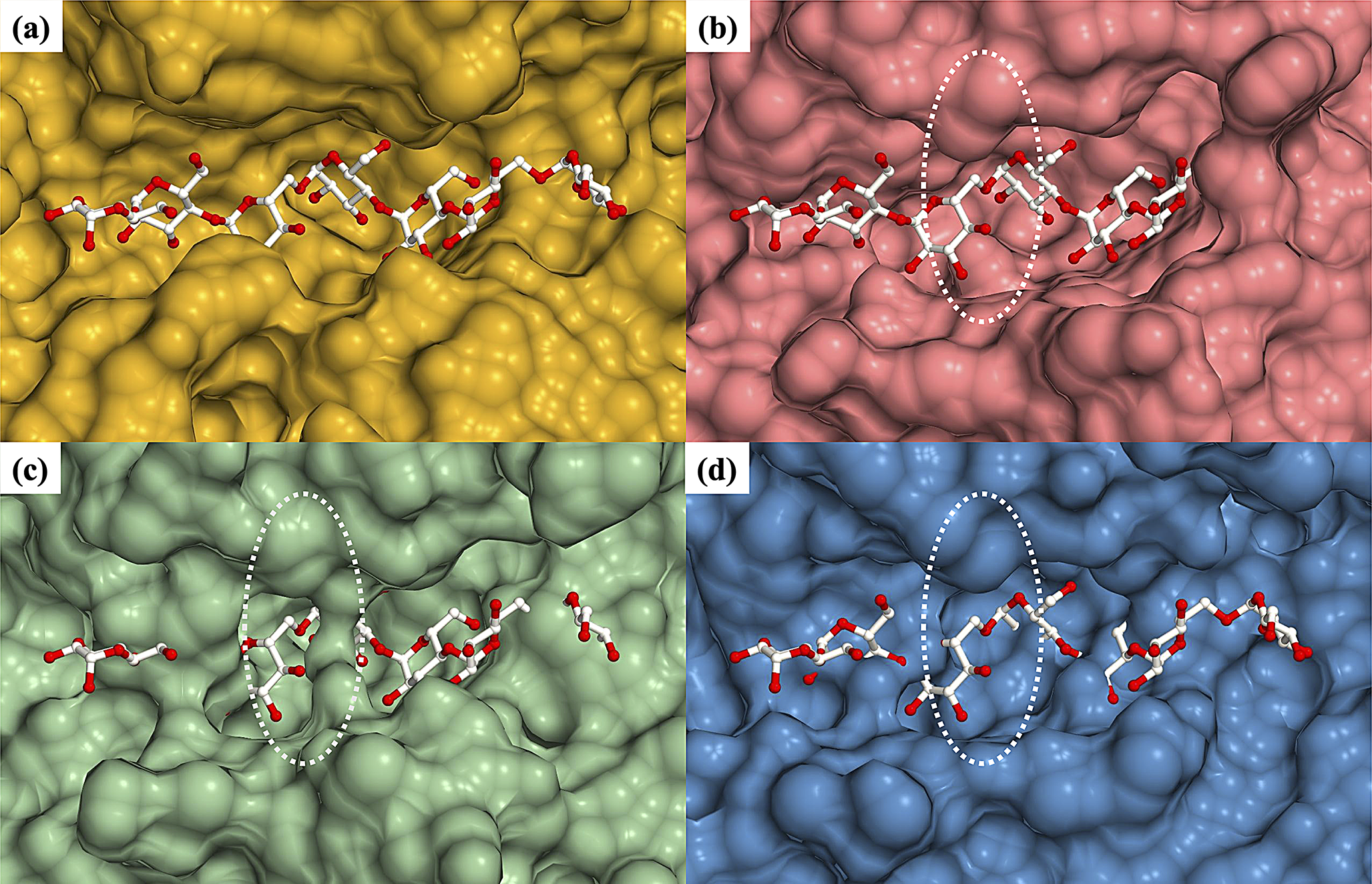

Supplement: Supplementary file 6 — Supplementary Material 3 [file 11103_2025_1623_MOESM6_ESM.tiff]

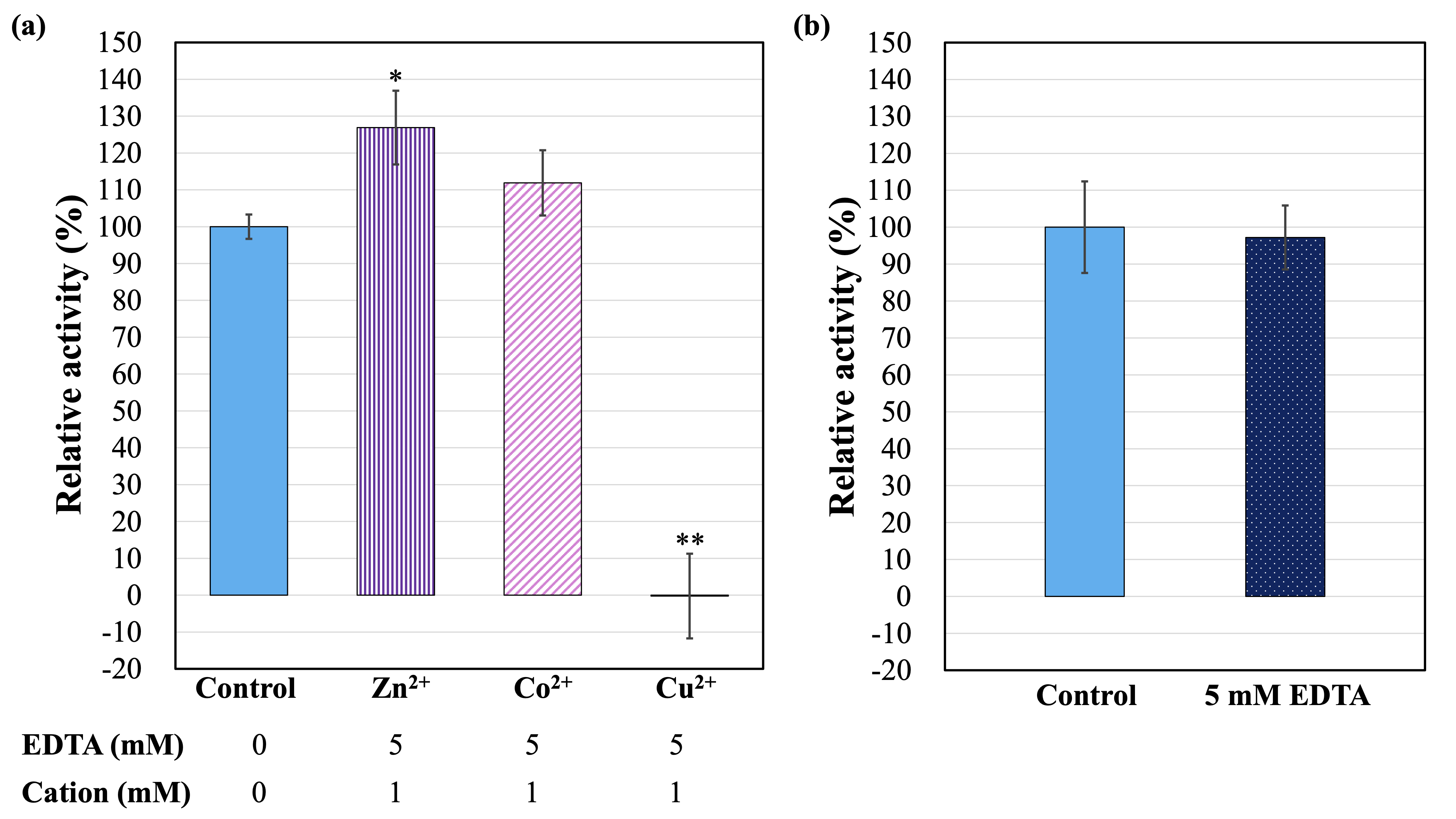

Supplement: Supplementary file 7 — Supplementary Material 3 [file 11103_2025_1623_MOESM7_ESM.tiff]

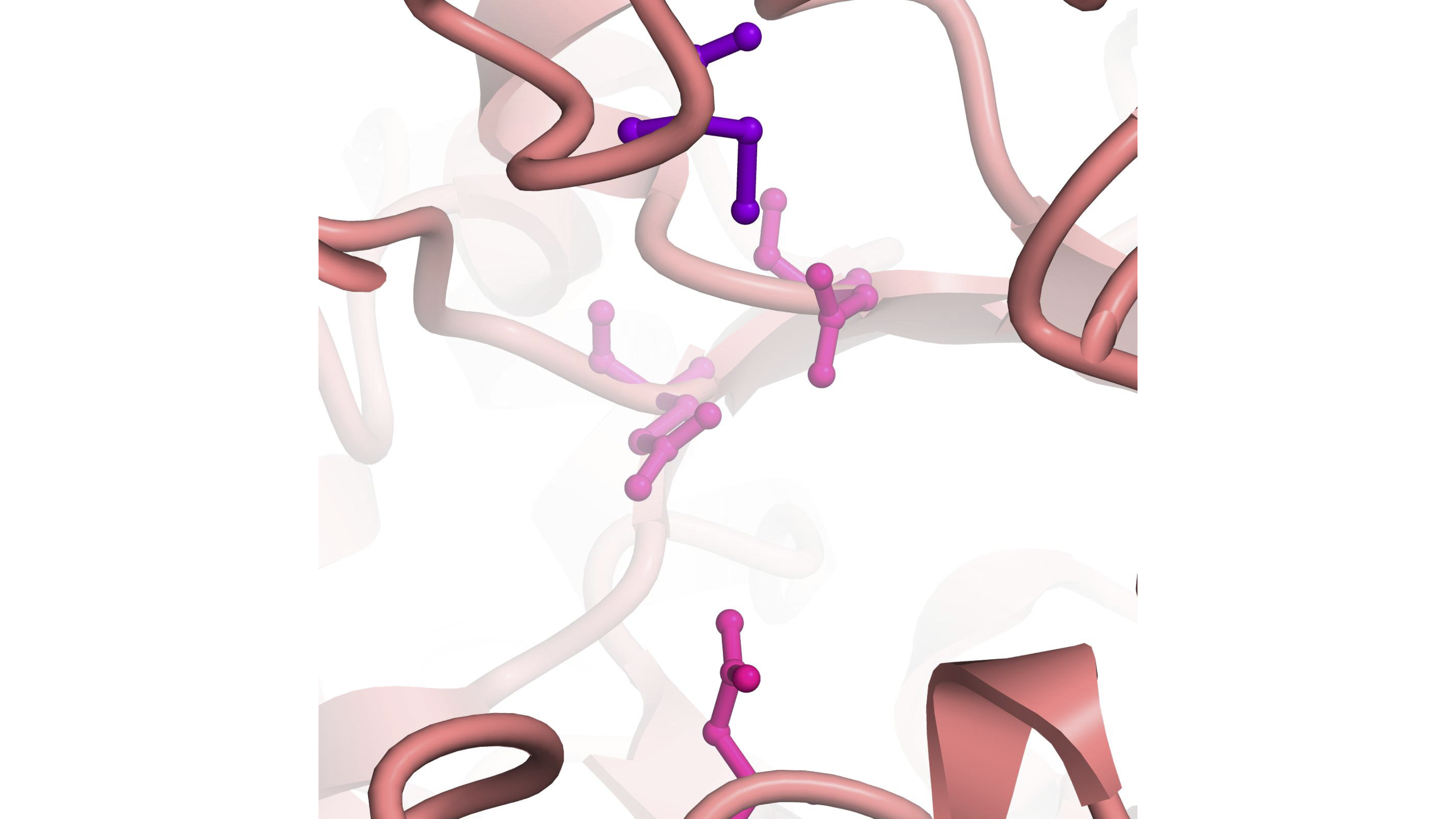

Supplement: Supplementary file 8 — Supplementary Material 3 [file 11103_2025_1623_MOESM8_ESM.tiff]

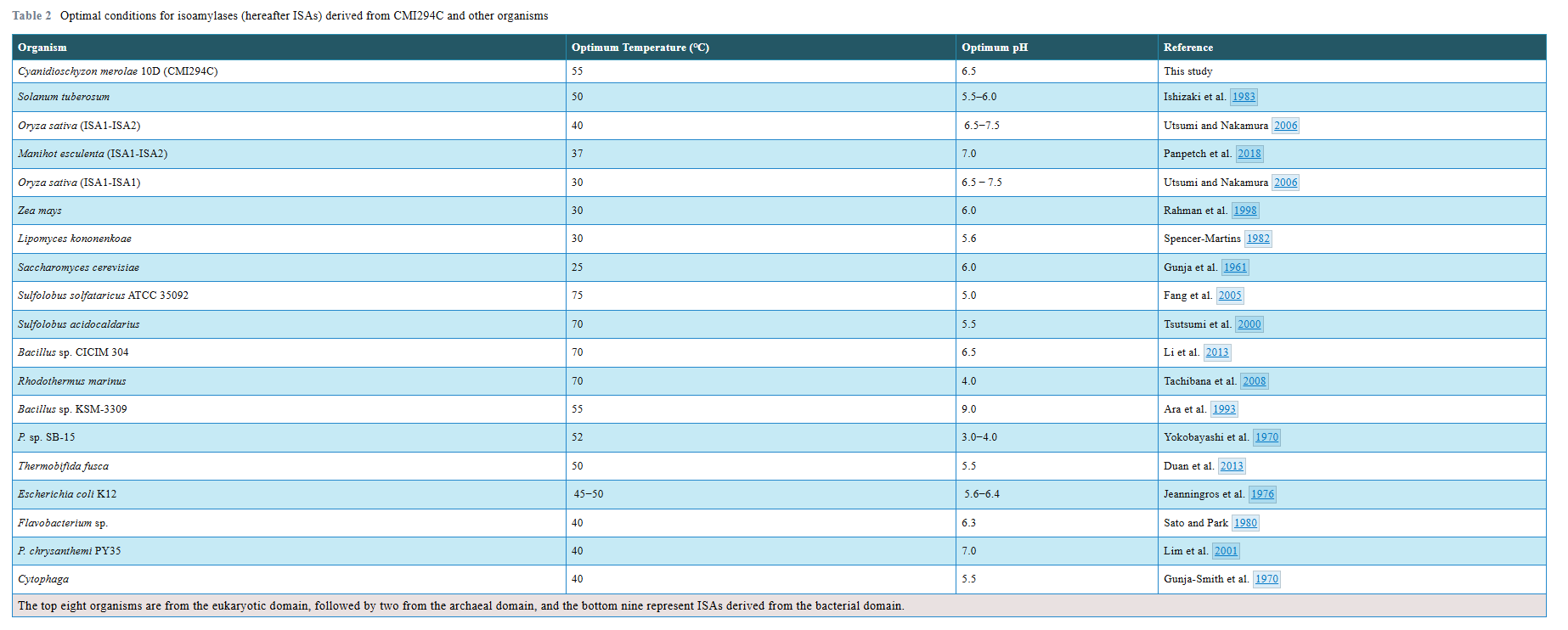

Supplement: Supplementary file 9 — Supplementary Material 3 [file 11103_2025_1623_MOESM9_ESM.odt]

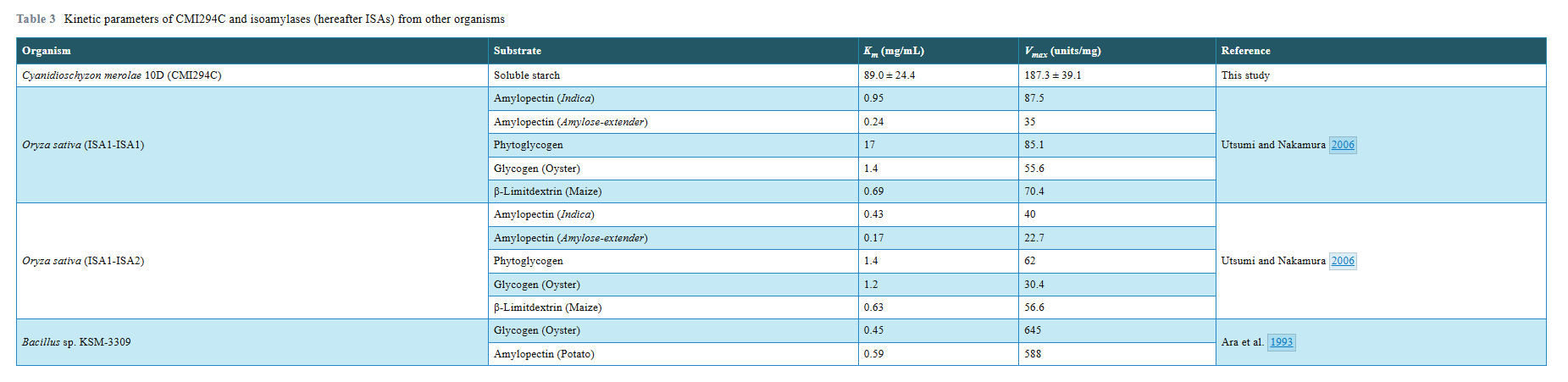

Supplement: Supplementary file 10 — Supplementary Material 3 [file 11103_2025_1623_MOESM10_ESM.odt]

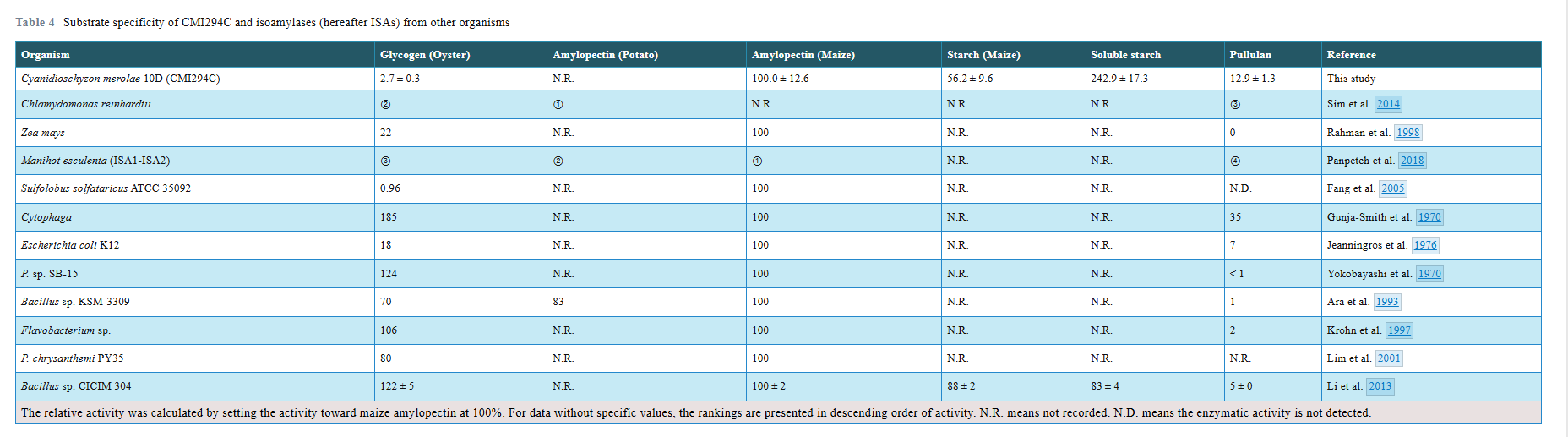

Supplement: Supplementary file 11 — Supplementary Material 3 [file 11103_2025_1623_MOESM11_ESM.odt]

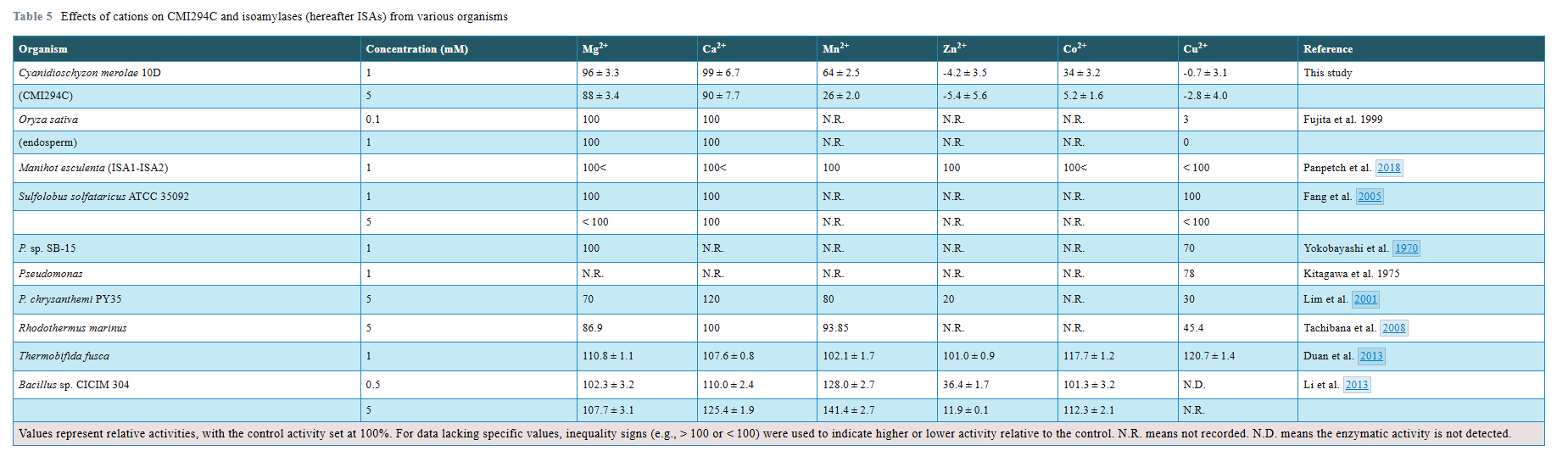

Supplement: Supplementary file 12 — Supplementary Material 3 [file 11103_2025_1623_MOESM12_ESM.odt]
