# Supplementary material for: Superior thermostability and divalent cation sensitivity of isoamylase CMI294C from Cyanidioschyzon merolae
Source: Plant Mol Biol. 2025 Jul 31;115(4):99. doi: 10.1007/s11103-025-01623-4 (PMC12313807; doi:10.1007/s11103-025-01623-4)
Supplement: Supplementary file 13 — Supplementary Material 3 [file 11103_2025_1623_MOESM13_ESM.docx]

**Supplemental Figure Legends**

**Fig. S1** CMI294C activity in the presence of EDTA or inhibitory cations. Each point represents the mean, and error bars indicate the standard deviation (n = 3). Activities are expressed relative to the control activity, which is set as 100%. **P* < 0.05, ***P* < 0.005 (a) CMI294C activity with 5 mM EDTA and 1 mM inhibitory cations. (b) CMI294C activity with 5 mM EDTA.

**Fig. S2** Positional relationship between the catalytic residue and Cys393 of CMI294C. All amino acid residues of CMI294C are shown as ribbon models. Only the catalytic residue and Cys393 are displayed as stick models, with the catalytic residue colored deep pink and Cys393 colored purple. The 3D model was prepared using CueMol2.
